# Quality assurance in proton beam therapy using a plastic scintillator and a commercially available digital camera

**DOI:** 10.1002/acm2.12143

**Published:** 2017-07-29

**Authors:** Mansour Almurayshid, Yusuf Helo, Andrzej Kacperek, Jennifer Griffiths, Jem Hebden, Adam Gibson

**Affiliations:** ^1^ University College London Medical Physics and Biomedical Engineering London UK; ^2^ Clatterbridge Cancer Centre Medical Physics and Biomedical Engineering London UK

**Keywords:** proton therapy, quality assurance, radiotherapy

## Abstract

**Purpose:**

In this article, we evaluate a plastic scintillation detector system for quality assurance in proton therapy using a BC‐408 plastic scintillator, a commercial camera, and a computer.

**Methods:**

The basic characteristics of the system were assessed in a series of proton irradiations. The reproducibility and response to changes of dose, dose‐rate, and proton energy were determined. Photographs of the scintillation light distributions were acquired, and compared with Geant4 Monte Carlo simulations and with depth‐dose curves measured with an ionization chamber. A quenching effect was observed at the Bragg peak of the 60 MeV proton beam where less light was produced than expected. We developed an approach using Birks equation to correct for this quenching. We simulated the linear energy transfer (LET) as a function of depth in Geant4 and found Birks constant by comparing the calculated LET and measured scintillation light distribution. We then used the derived value of Birks constant to correct the measured scintillation light distribution for quenching using Geant4.

**Results:**

The corrected light output from the scintillator increased linearly with dose. The system is stable and offers short‐term reproducibility to within 0.80%. No dose rate dependency was observed in this work.

**Conclusions:**

This approach offers an effective way to correct for quenching, and could provide a method for rapid, convenient, routine quality assurance for clinical proton beams. Furthermore, the system has the advantage of providing 2D visualization of individual radiation fields, with potential application for quality assurance of complex, time‐varying fields.

## 1 INTRODUCTION

Scintillator‐based detectors are used in many ionizing radiation‐based imaging modalities. Recently, there has been an increase in research on characterizing and evaluating plastic scintillators for use as dosimeters for quality assurance (QA) applications.^1^, ^2^, ^3^ An ideal dosimeter should provide precise and accurate measurements; it should have a well‐described dose**–**response and be able to correctly resolve a depth‐dose curve, while also reliably tracking fast changes in dose.

Beddar et al.^4^ showed that when a plastic scintillating fiber attached to a photomultiplier tube was exposed to a photon beam, it exhibited high sensitivity, a linear dose**–**response, a fast response to ionizing radiation and low angular dependence. In the same paper, plastic scintillators were also found to have the best energy independence compared to other dosimeters used in radiotherapy. Their output is also independent of pressure and temperature.

With the proton radiotherapy field growing rapidly, there is a need for fast and accurate dosimetry tools. Many dosimeters have been reported for use in clinical proton dosimetry such as ionization chambers and ionization chamber arrays. However, 2D arrays of ionization chambers are not widely used although they provide fast measurements, because a high spatial resolution is required to measure the sharp fall in depth dose at the end of the protons' range, the protons spot shape, and the profile of a narrow proton beam.^5^, ^6^ Scintillators' properties appear to make them viable for use in proton quality assurance. The scintillator can be either small (e.g., scintillation fibers arranged in 2D arrays) or large (e.g., liquid or plastic scintillators). For instance, the response of a single scintillating fiber (i.e., containing scintillating solutes and surrounded with a nonscintillating optical fiber) in a proton beam was evaluated, and it was found that the scintillating of the clear optical fiber led to undesired noise. Several features of large scintillators enable them to be good candidates for dosimetric measurements of proton beam such as fast response and high spatial resolution.^7^ Recently, large 3D‐volume liquid scintillator detectors were used to verify proton range and position for scanned proton beams and showed that they were able to provide precise position results within 0.7% and an accuracy in proton range to within 0.3 mm on average.^8^, ^9^ However, the materials making up a liquid scintillator are not suitable for hospital environments due to toxicity and the need to deoxygenate the scintillator prior to use to optimize the light output.^10^ Furthermore, the emitted light of the liquid scintillator may be reflected or refracted at the walls of the tank causing unwanted light signals.^9^, ^11^ Plastic scintillators have attractive features for some practical applications because they are nontoxic, robust and, durable, and there is no risk of leaks.

For an ideal dosimeter, the light output should be proportional to the energy deposited. However, this is not the case if a plastic scintillator is irradiated with charged particles with high linear energy transfer (LET) such as protons.^12^ In this case, the light output is suppressed in a process known as quenching. This effect is well known and is described by Birks Law.^13^ As protons slow down due to the energy loss (*dE/dx*), more energy is transferred to the medium (higher LET). However, a greater proportion of energy is lost to interactions which do not emit light, hence the relative light output (*L*) is reduced in the single Bragg Peak and at the end of the Spread‐Out Bragg Peak (SOBP). In eq. (1), kB is Birks constant (mm MeV^−1^), which depends on the charged particle type and the scintillation material, and L_0_ is the scintillation efficiency.^14^, ^15^

(1) $$\frac{\mathrm{dL}}{d}=\;L_{0}\frac{\frac{\mathrm{dE}}{\mathrm{dx}}}{1+kB.\frac{\mathrm{dE}}{\mathrm{dx}}}$$

Several studies have used Birks equation to estimate Birks constant in order to calculate the quenching of the measured scintillation data.^16^, ^17^, ^18^ Recently, Robertson et al.^3^ conducted a study using a liquid scintillator in proton therapy to correct for quenching of the scintillation light produced by 86.60 to 161.6 MeV protons. The Birks constant was estimated and used to analytically correct the measured scintillation results. The height of the Bragg peak for the corrected measured scintillation agreed to within ±10% of the depth‐dose profile calculated from Monte Carlo simulation (MCS) using MCNPX. In our study we extend Robertson et al.'s work by simulating the expected quenched scintillation and ideal scintillation light in a plastic scintillator for proton beams over an energy range of 38.94–60 MeV and generating correction factor using Geant4 MCS.^19^, ^20^, ^21^ This technique makes the system more applicable, and gives an easy method to provide a quenching correction at any given beam energy.

In this article, we propose a water‐equivalent detector system that uses a commercial camera to photograph a large volume plastic scintillator for the dosimetry of protons. This low‐cost, convenient, clinically achievable system extends previous work in proton radiotherapy by using a large solid plastic scintillator, a commercial camera and a completely numeric technique for quenching correction. To our knowledge, neither the use of a plastic scintillator larger than the beam field size, nor a numeric quenching correction, has been reported previously. We investigate the system's response to changes in energy and dose rate, and compare the depth‐dose measured with the scintillation detector to that measured with an ionization chamber. Finally, we propose and characterize a method for simulating and correcting the quenching effect using a Geant4 simulation package that can track all photons generated inside the scintillator.^19^, ^22^, ^23^

## 2 MATERIALS AND METHODS

### 2.A Measurement system

#### 2.A.1 Detector system setup

Proton irradiations were performed using the cyclotron at the Clatterbridge Cancer Centre. The cyclotron produces a 62 MeV proton beam delivered by a fixed beam line; the proton energy is 60.0 MeV at the treatment isocenter, 7 cm in air from the collimator nozzle. A range modulator consisting of 0.84 mm stepped thicknesses of poly‐methyl methacrylate (PMMA) is usually placed in the beam to treat patients with eye tumors.^24^

**Figure 1:**
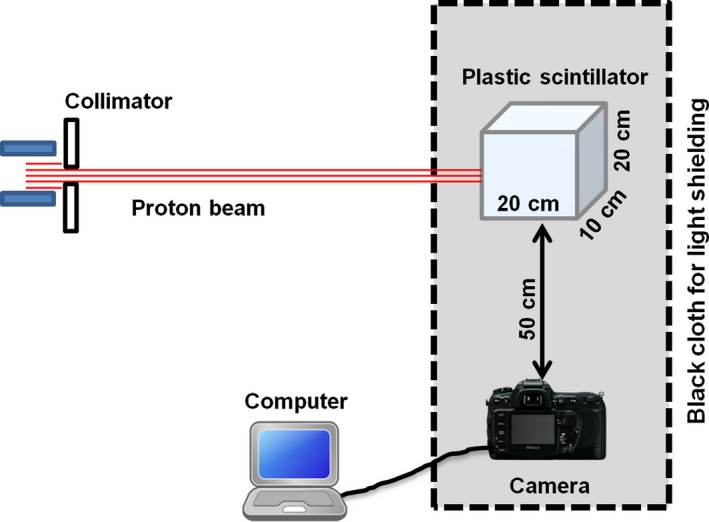
Schematic of the experimental setup. The camera was positioned at 50 ± 0.5 cm perpendicular to the proton beam and data acquisition was performed using a Nikon camera controlled by software on a laptop computer situated outside the treatment room connected via a 25 m USB cable. Black cloth and tape were used to exclude ambient light.

The two main components of the scintillator detector system are the plastic scintillator where the deposited proton energy is converted into light, and the camera that captures images of the emitted light which are subsequently analysed by a computer. A schematic overview of the prototype scintillator detector system is shown in Fig. 1.

The BC‐408 plastic scintillator (20 cm × 20 cm × 10 cm) selected for this study (Table 1) was exposed to the proton beam and the camera was used to photograph the emitted light. The front surface of the scintillator was positioned at the treatment isocenter, 7 cm from the proton nozzle. The camera used was a Nikon D7100 camera with a Nikon AF‐S DX NIKKOR 35 mm f/1.8G lens. It was mounted on a tripod. The camera uses a complementary metal‐oxide‐semiconductor (CMOS) sensor which has 6034 × 4024 pixels (24 mm × 16 mm) with ~4 μm pixel size and 14‐bit dynamic range.

**Table 1:** The properties of BC‐408 plastic scintillator.^29^

Parameter	BC‐408
Core material	Polyvinyltoluene
Refractive index	1.58
Density, g/cm^3^	1.03
Emission peak (*λ*),nm	425
No. of photons/MeV	~8000
Ratio H:C Atoms	1.104
Light attenuation length, cm	210

The camera exposure settings were selected by (a) choosing the lowest ISO setting to minimize noise in the image; (b) setting the aperture so that the depth of field covered the field size of the proton beam; and (c) setting the exposure time such that the dynamic range of the camera was filled without reaching saturation at the highest light output. These settings were kept constant for all images. The relative pixel size in the final image was measured by imaging a ruler placed along the central axis of the beam.

#### 2.A.2 Image corrections and analysis

Unwanted signals can reduce the accuracy of the measurements and so should be removed from the raw data where possible. First, the level of the dark signal due to electronic interference from the cyclotron and delivery system was determined by acquiring a photograph with the beam on, but in the absence of the scintillator. Then, a scattering water‐equivalent material (paraffin wax of a similar size to the scintillator, wrapped in black cloth) was placed in the beam to determine the effect of scattered nonoptical emissions on the detected signal. Background images were then subtracted where appropriate. A median filter was applied to the image.

The mean image intensity with the beam on but without a scatterer present was 2.3 times that with the beam off. When the wrapped scatterer was present, the background was 2.7 times that with the beam off. However, the total background count was still less than 1% of the signal. Despite there being no substantial impact from background on the measured signal, for completeness, we still correct for background in our data.

Cerenkov emission, which is light emission due to charged particles travelling at relativistic speeds through a medium, is another possible noise source. A 60 MeV proton beam is not capable of producing Cerenkov emission directly.^25^, ^26^ However, nuclear decay and nonelastic nuclear interactions can still create some Cerenkov light emission. Previously, we used Geant4 to determine the average light production of scintillation and Cerenkov emission when the scintillator was irradiated by a 60 MeV proton beam.^25^, ^26^ We found the relative production of Cerenkov emission compared to scintillation light was of the order 10^−5^. Therefore, we neglected the contribution of Cerenkov light emission in this work.

Vignetting is a lens effect that reduces the brightness at the edge of the image. It is caused by geometrical effects within the lens.^3^, ^25^, ^26^ A vignetting correction was performed by acquiring a flat white image for a homogeneous light field supplied by a lightbox trans‐illuminating a homogeneous scattering medium. The correction was obtained experimentally for each pixel.

Another image distortion effect occurs due to magnification as we have a 3D light field within the scintillator projected onto a 2D image plane. The geometry and intensity of projections depend on the field size of the proton beam and the distance between the camera and the beam. Photographs of the light output show the integrated light is emitted predominantly from a 2D plane near the camera, but we want to extract the light along the central axis. Therefore, it is necessary to consider magnification to be able to compare the ionization chamber measurement, and the simulated scintillation light, to the measured light.

The parameters that contribute to the magnification are the distance between a given projection and the midline projection, *x*, the depth of the midline projection, *y*, and the distance between the midline projection and the camera, *r*. Light generated near to the camera (i.e., +1.25 cm from the midline for a 2.5 cm field size) contributes a higher intensity than light generated from behind the midline. We adopted the method used by Helo et al.^25^, ^26^ to correct for this. The intensity I′ of the image of the given projection at distance *x* given the intensity of the image of the midline projection, I, estimated by the measurement can be found using eq. (2):

(2) $$I^{\prime }=\left(\frac{r^{2}}{{\left(r\pm x\right)}^{2}}\right)I$$

Equation (3) can be used to predict the relative magnification effect (y′):

(3) $$y^{\prime }=\left(\frac{r\pm x}{r}\right)y$$

**Figure 2:**
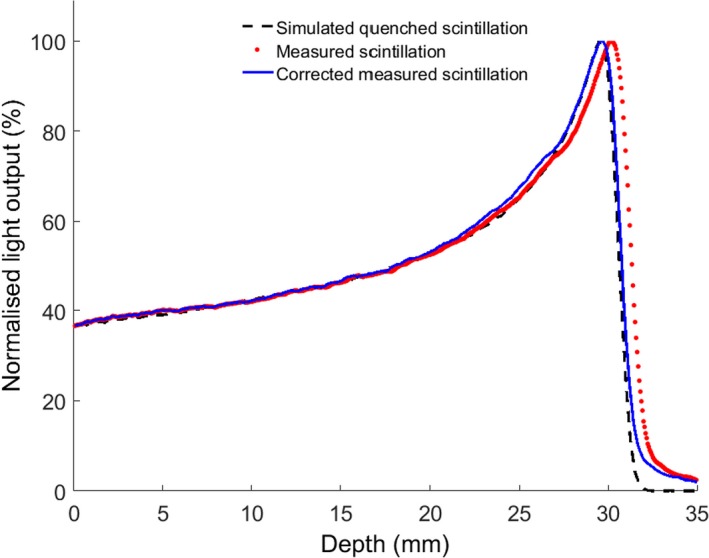
Percentage depth doses shown for data extracted from the photography directly without magnification correction (red dots), simulated light output with quenching (black dashes) and the measured data after correction for magnification (blue line). The agreement between data and simulation improves following the magnification correction.

Using eqs. (2) and (3), the intensities can be calculated across the beam. The measured scintillation light distribution along the central axis can then be compared to the simulated scintillation distribution as shown in Fig. 2.

Through the measurements, three repeated uncompressed raw images were acquired during each irradiation of the scintillation material. Following this, conversion of the raw image data to relative dose distribution was a multistep process. The first step was to convert the raw images from Nikon's proprietary .NEF format to a format that can be read by Matlab (The Mathworks Inc., USA) using dcraw which is an open source software.^27^ An uncompressed .tiff was used to avoid degradation of the image quality. To avoid any systematic deviations, the dark image acquired pre‐irradiation was subtracted from the images obtained during the irradiation and corrected for magnification and vignetting. A region of interest (ROI) was selected and the same ROI was used through all of the measurements. Then the ROIs of the three images acquired when the beam was on were combined to obtain a cumulative light intensity. The mean and the standard deviation of the combined ROI were then calculated.

### 2.B Quenching correction

#### 2.B.1 Comparison of scintillation vs ionization chamber measurements

The scintillation light imaged using the camera was compared to measurements taken with an ionization chamber calibrated for use with the Clatterbridge 60 MeV proton beam. The depth‐dose curve was measured along the central axis in the plastic scintillator with a 2.5 cm diameter beam at a source‐to‐surface distance (SSD) of 7 cm, and compared to a depth‐dose curve measured with a parallel‐plate ionization chamber (Markus, PTW, Freiburg, Germany) using the 60 MeV proton beam. Two modes were used to acquire the images: the first was a pristine 60 MeV Bragg peak; the second was a fully spread out Bragg‐peak (SOBP) achieved by placing a modulator in the beamline. For the pristine Bragg peak mode, 1 monitor unit (MU) corresponds to 0.9 Gy at the Bragg peak whereas for the SOBP mode, 1 MU corresponds to 0.7 Gy at the centre of modulation.

#### 2.B.2 Monte Carlo simulation

An approach has been developed in this work for correction of quenching using MCS in Geant4 version 10.0.^19^ First, the response of the scintillator to the proton beam was modeled. Each dimension of a 20 cm × 20 cm × 10 cm block of the BC‐408 plastic scintillator was divided into 800 equal voxels, giving a voxel size of 0.25 mm × 0.25 mm × 0.125 mm. The material properties of the scintillator were taken from the manufacturer's data sheet (Table 1). The refractive index of the air and scintillator were assumed to be 1 and 1.58 respectively. The total light yield and light attenuation length were included and are listed in Table 1. The emission spectra of the BC‐408 scintillator were included in the MCS with a spectral resolution of 10 nm. Photons were scored in a pixel if they were generated in that pixel; photons travelling through the scintillator were neglected.

The primary beam consisted of 10^6^ incident protons, with the energy of the beam assumed to be a Gaussian distribution with a mean of 60 MeV and standard deviation of 0.36 MeV. A circular collimator of diameter 2.5 cm was used to collimate the beam. Prior to entering the scintillator, the protons passed through 7 cm of air. The QGSP_BIC_EMY physics list class was used, (this is the reference physics list recommended for the simulation of hadron therapy applications^28^), with additional lists to model optical processes such as boundary interactions (i.e., G4OpBoundaryProcess) and scintillation (i.e., G4Scintillation) including Birks law (i.e., G4EmSaturation).

#### 2.B.3 Determination of Birks constant

As protons penetrate a scintillator, the LET of the incoming proton beam increases nonlinearly, leading to a reduction in the expected light output due to the quenching effect. If kB is 0, no quenching effect would be shown and *dL/dx* would be directly proportional to the LET. However, for high LET particles, kB>0 and *dL/dx* is non‐linear with light output, as indicated in eq. (1).

In order to obtain kB in eq. (1) for implementation into the simulation, LET was modeled for in Geant4 although an alternative approach would be to measure the LET values by averaging the collision energy deposited over a finite trajectory length as described by Guan et al.,^29^ along with the depth dose curve and measured scintillated light output. The current model neglects any ionization that comes from secondary particles.

#### 2.B.4 Calculation and comparison with measurements

**Figure 3:**
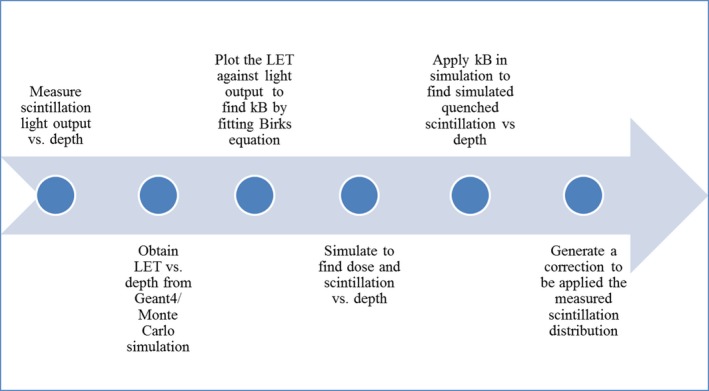
Summary of the quenching correction procedure.

The average energy deposited in the scintillator was scored in Geant4 alongside the scintillation light generated. Knowledge of kB allows the scintillation light distribution to be scored both with and without the influence of quenching. The outcomes from the simulation were the depth‐dose profile, scintillation light distribution in the absence of quenching, and quenched scintillation light distribution. The simulations of ideal and quenched scintillation distributions allowed generation of a correction which was applied to the measured distribution to produce the corrected scintillation output. A summary of the correction procedure is shown in Fig. 3.

### 2.C Detector system characterization

The results presented in this section, showing tests of measured scintillation, were corrected for quenching after obtaining the correction factor for the scintillation light vs depth.

#### 2.C.1 Reproducibility

The reproducibility of the system was tested by irradiating the scintillator multiple times under identical irradiation parameters with a 60 MeV proton beam (4.5 Gy at 18 Gy/min).

#### 2.C.2 Dose linearity

To assess the linearity of the dose**–**response, the scintillation light output distributions were imaged at a range of doses between 0.45 and 9 Gy. Images were taken with a 25 mm collimator, at a constant dose rate of 18 Gy/min.

#### 2.C.3 Scintillation light variation at different dose rates

The output of a clinical detector system should be independent of dose rate. Images were acquired using five dose rates to deliver 4.5 Gy at dose rates from 4.5 to 36 Gy/min in order to test this.

#### 2.C.4 Scintillation light output at different energies

In order to study the scintillator response to proton beam energy, different thicknesses of PMMA were placed in the path of the 60 MeV proton beam, and the generated scintillation light imaged. Software developed by Meroli^30^ was used to calculate the energy loss in the PMMA for each thickness to estimate the proton energy. The scintillation light depth profile was measured as a function of the proton energy. The pixel intensities were summed and then plotted against beam energy. The relation is expected to be linear at beam energies greater than 4 MeV. Below this energy, the relationship is nonlinear.^15^ It is expected that the relationship would be linear for all energies after correcting for quenching effect.

## 3 RESULTS

### 3.A Scintillation vs ionization chamber measurements

**Figure 4:**
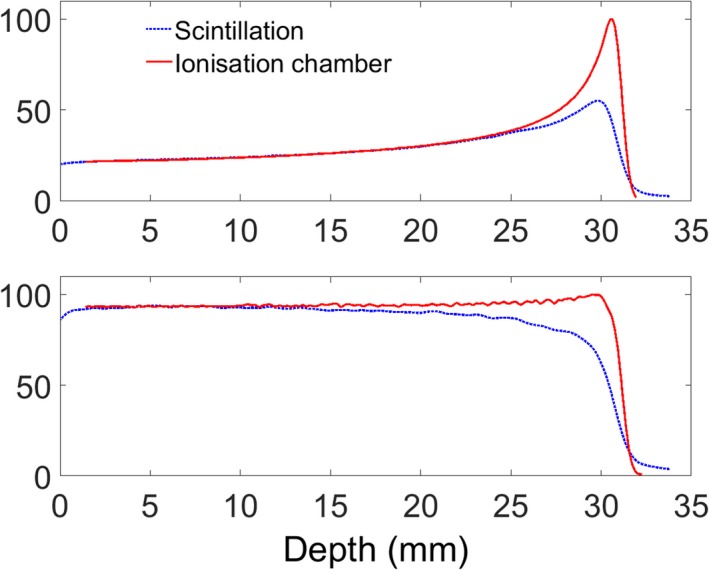
Depth‐dose profile comparisons between ionization chamber measurement and measured scintillation light for a pristine Bragg peak (top) and a spread out Bragg peak (bottom).

Relative depth‐dose curves of a pristine Bragg peak and SOBP measured with both an ionization chamber and the scintillator‐based detector system are given in Fig. 4. There is an obvious reduction in the Bragg peak measured by the scintillator, reflecting the quenching effect. The Bragg peak is reduced to 2.8 times the plateau height in the scintillator measurements compared to 5.1 times the plateau when measured by the ionization chamber. Another difference between the distributions is the small tail occurring at 5.1% of the peak intensity at the end of the distal fall off of the scintillator data. This tail has been reported in the literature. Its source is uncertain, but it is approximately exponential and has been attributed to scattering of scintillation photons generated due to absorption and re‐emission when traveling through the scintillator.^3^

### 3.B Determination of Birks constant

**Figure 5:**
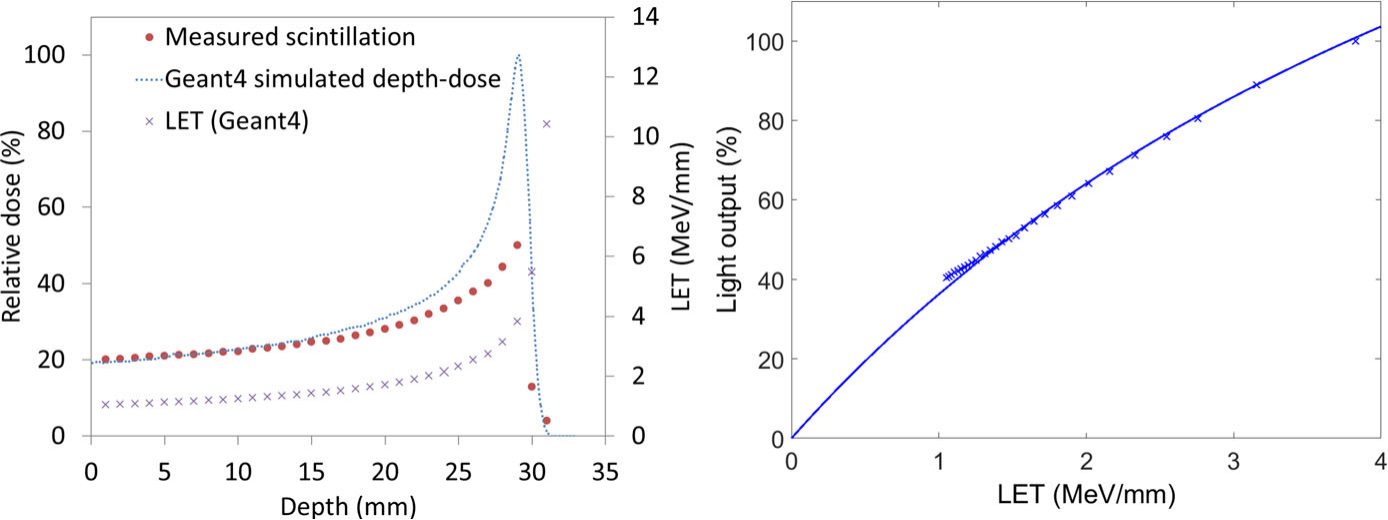
(a) LET values simulated by Geant4, depth dose profile simulated in Geant4, and the measured scintillation light distribution as a function of depth for a 60 MeV proton beam in a BC‐408 scintillator (b) The measured scintillation light vs simulated LET for the 60 MeV proton beam (note that the highest LET points do not contribute to light output as they occur beyond the Bragg peak).

Figure 5(b) shows the measured scintillation light output plotted against LET, and demonstrates the nonlinearity due to quenching. Equation (1) was fitted to the data in Fig. 5(b) using the “NonlinearLeastSquares” option in the “fit” routine in Matlab R2014a, and Birks constant, kB, was found to be 0.154 mm MeV^−1^ (95% confidence level at 0.137 to 0.170 mm MeV^−1^). To our knowledge, this is the first report of kB for the BC‐408 plastic scintillator and it lies within the range of published values of kB for a closely related BC‐400 plastic scintillator which vary from 0.124 mm MeV^−1^ to 0.207 mm MeV^−1^.^16^, ^17^ To validate this, LET was also calculated in SRIM, an online software package,^31^ and found to be 0.147 mm MeV^−1^ (95% confidence 0.133 to 0.160 mm MeV^−1^), which is in good agreement with the Geant4 results beyond the Bragg peak, where we have noticed that SRIM can slightly underestimate the LET value due to the mono‐energetic input of the primary beam.

### 3.C Quenching correction

**Figure 6:**
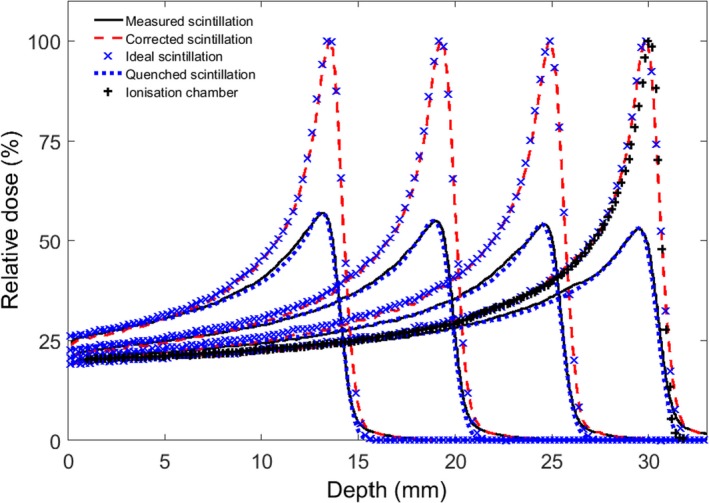
Percentage depth dose curves at 38.94, 46.77, 53.86, and 60.00 MeV, showing (a) the simulated depth dose (blue crosses); (b) the depth dose directly measured by the camera (solid black line); (c) the simulated quenched light output (blue bars); and (d) the measured light output after correction for quenching (red dashes).

Figure 6 shows percentage depth dose curves for beams with energies of 38.94, 46.77, 53.86, and 60.00 MeV, and ionization chamber measurements for the 60 MeV beam. For each energy, four plots are shown: (a) the simulated depth dose (blue crosses); (b) the depth dose directly measured by the camera (solid black line); (c) the simulated quenched light output (blue bars); and (d) the measured light output after correction for quenching (red dashes). As expected, the measured light output after correction for quenching agrees with the simulated depth dose.

For the 60 MeV proton beam, the simulated Bragg peak range (i.e., the 90% distal dose point) and the range taken from the corrected measured scintillation both agree with the range measured from the ionization chamber (black crosses), with 0.2 mm accuracy and 3% accuracy for the peak/plateau ratio.

### 3.D Reproducibility

Depth‐dose profiles were extracted from the photographs and corrected for the quenching effect. All results below refer to measured profiles after the quenching correction was applied. The results indicate that the system was stable as the variation in the average light output of seven repeated results was found to be less than 0.80%.

### 3.E Dose linearity

**Figure 7:**
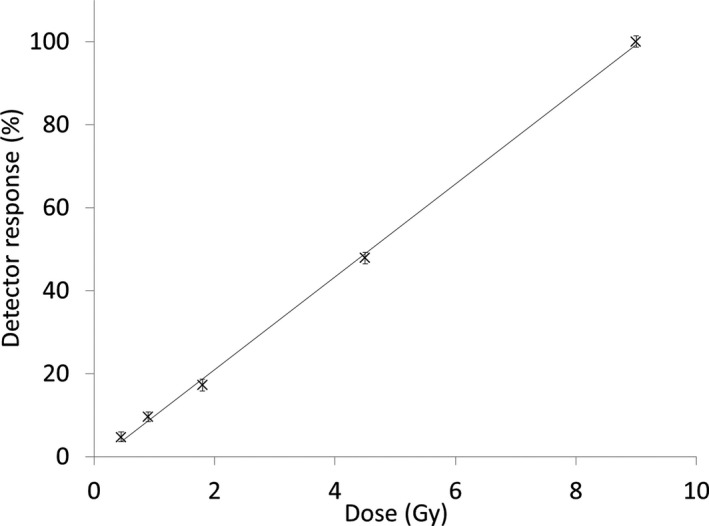
Linearity of the scintillation detector system as a function of dose for a 60 MeV proton beam.

The dose**–**response relation of the detector was checked by delivering different doses to the scintillator and imaging the emitted light. Figure 7 demonstrates that the system is linear with dose.

### 3.F Scintillation light variation with dose rate

**Figure 8:**
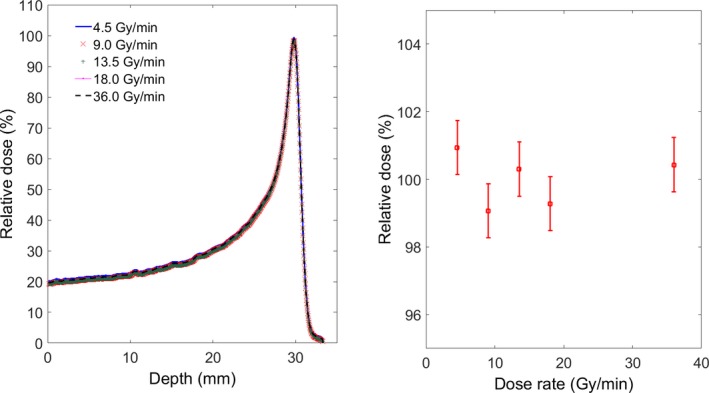
The response of the scintillation detector system after delivering 4.5 Gy at different dose‐rates for a 60 MeV proton beam. Vertical bars on the right hand graph give the measurements error.

Results from the dose rate dependency test are displayed in Fig. 8. The measurement uncertainties, assessed by measuring the standard deviation relative to the mean at different dose rates, were less than 1%.

### 3.G Variation in scintillation light with proton beam energy

**Figure 9:**
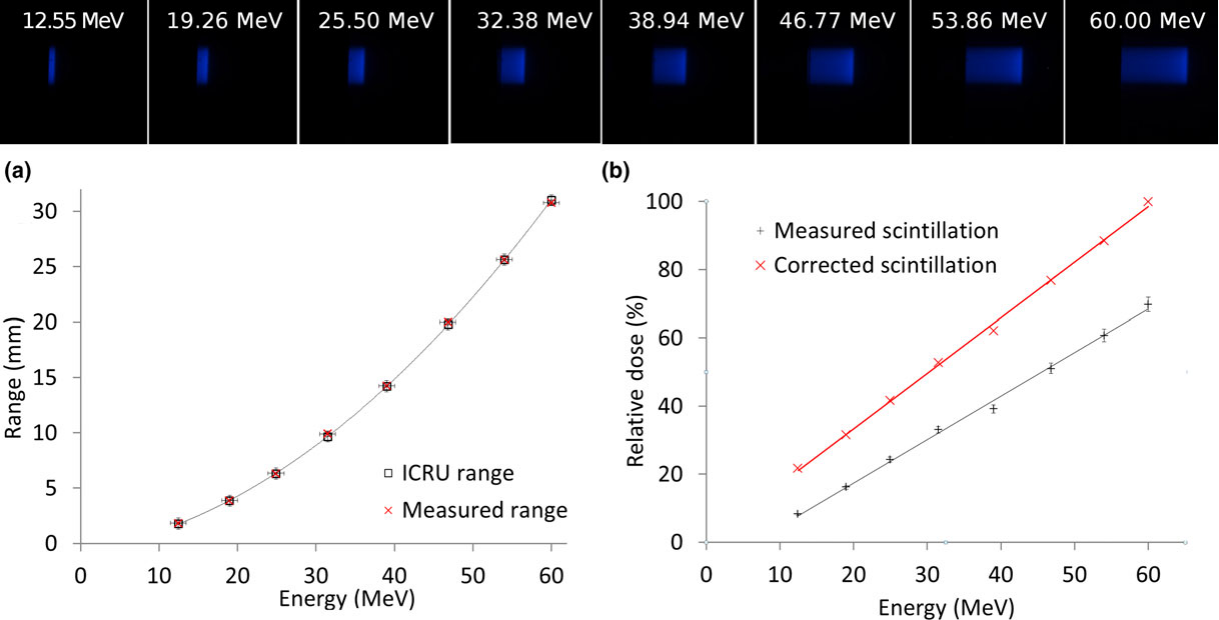
Images obtained by the scintillation detector system show (a) The variation in range with the proton energies used in this study (b) The impact of different proton beam energies on the response of the detector system.

Figure 9 shows the measured range in the scintillator for eight different proton beam energies achieved by placing varying thicknesses of PMMA in the beam. The fit shows that the system responds linearly. The measured proton ranges in plastic scintillator were compared to the range data from ICRU^32^ and the agreement was found to be very good with a maximum difference of 0.16 mm.

## 4 DISCUSSION

A prototype system for proton therapy QA has been designed using a plastic scintillator and a commercial camera. The characterization results for this prototype system show excellent linearity. In addition, the system shows reproducibility of results of around 0.8%. The results are also independent of the applied dose‐rate to within 2%. These variations could be attributed to either the instability of the cyclotron or the time integrated by the camera.

Background signals, especially from scattered radiation, have an impact on the accuracy of the camera measurements. The contribution of these signals to the measurements is minimal, but depends on the geometry of the experimental set‐up, the distance between the camera and the beam line and energy. A possible solution could be to shield the camera, which we will attempt for future work; however, it will remain good practice to obtain background measurements before any series of acquisitions. The issue of magnification was solved analytically (Fig. 2) and showed very good agreement with measurement.

To predict the kB factor, it was essential to measure the light output because the scintillation light output and the quenching effect depend on the type of scintillator (i.e., plastic, liquid or scintillating fibers). The flexibility of a Monte Carlo simulation allows bespoke solutions for realistic clinical beams, any scintillator composition and more complex beam geometries than an analytical formulation. Our results are promising and indicate that the quenching problem can be solved effectively by Geant4 simulation, allowing the system to be used for relative dosimetry in proton therapy. Nevertheless, optical artifacts such as internal light scattering, magnification, and blurring require further consideration in order to enhance the peak/plateau ratios and the appearance of the Bragg peak tail.

To progress the system to a point where it is ready for translation into daily use in a clinical setting, some further points will need to be addressed such as how we translate this system to a larger field size, wider energy range, and SOBP.

For a larger field size with a passive scattering system, the magnification effect will increase. We expect that the analytical method we have used in this work to correct for the magnification effect could be applied for clinically relevant field sizes. In more complex cases, such as use of multileaf collimators, very large field sizes, or if nonlinear optical propagation such as attenuation becomes significant, an equivalent correction factor could be obtained numerically. The system can also be used to measure transverse profiles.

The current system can be used for energies up to 175 MeV (~20 cm range in water), and this is limited only by the size of our scintillator. For higher energies a bigger scintillator could be used, or the scintillator currently used could be immersed in water to ensure the Bragg peak remains within the scintillator. At 60 MeV, the difference in the depth of the Bragg peak between water and the scintillator material in our simulation was approximately 0.25 mm, confirming the water equivalence of this scintillator.

The detector system can be used to correct SOBP beams for the quenching effect. By knowing the modulator wheel that has been used, we can simulate the SOBP, which consists of a sum of pristine Bragg peaks at different energies. The quenching effect can be calculated for each beam individually before weighted summing to form the SOBP. For pencil scanning beams, this detector system could be operated in video acquisition mode and offer advantages (fast and easy) over, for instance, an ionization chamber.

Our dose linearity and dose rate dependency measurements show that the system can determine the dose within 1% error, which is within required tolerances for much of radiotherapy QA.^33^ We have demonstrated the system gives reproducible results over short periods of up to a day, with an accumulated dose up to 140 Gy. Over longer periods, the reproducibility will be limited by radiation damage to the scintillator and the camera.

These features lead to a versatile system that can be used to do a rapid daily QA measurement for the prescribed depth. For example, we propose that images for a given energy and dose can be compared every morning to test the stability of the treatment machine. This could be quicker than scanning with a diode, with the advantage that the beam is visualized quickly in two dimensions with a photograph.

## 5 CONCLUSION

Despite the common use of scintillators, there is little data available for the correlation between dose and scintillation output, especially in proton therapy when using a camera for imaging. Here, we evaluate the dosimetric characteristics of a camera‐scintillation detector system for dosimetry of proton beams. The system has the advantages of providing a 2D view of dose distribution for individual radiation fields, while being fast, directly digital.^34^ Our results were found to be reproducible. However, the measured depth‐dose distributions using this system were lower than those measured with an ionization chamber due to the quenching effect occurring in the scintillator. We have proposed a method of correction for quenching, based on numerical rather than analytical methods, which shows promising results. It can be concluded that the detector system has the potential to be translated for use in quality assurance of clinical proton beams. Future challenges include 3D time‐varying data acquisition.

## CONFLICT OF INTEREST

The authors declare no conflict of interest.

## References

[1] Guillot M , Beaulieu L , Archambault L , Beddar S , Gingras L . A new water‐equivalent 2D plastic scintillation detectors array for the dosimetry of megavoltage energy photon beams in radiation therapy. Med Phys. 2011;38:6763–6774. 22149858 10.1118/1.3664007

[2] Lacroix F , Sam Beddar A , Guillot M , Beaulieu L , Gingras L . A design methodology using signal‐to‐noise ratio for plastic scintillation detectors design and performance optimization. Med Phys. 2009;36:5214. 19994531 10.1118/1.3231947 PMC2774352

[3] Robertson D , Mirkovic D , Sahoo N , Beddar S . Quenching correction for volumetric scintillation dosimetry of proton beams. Phys Med Biol. 2013;58:261–273. 23257200 10.1088/0031-9155/58/2/261 PMC3849813

[4] Beddar AS , Mackie TR , Attix FH . Water‐equivalent plastic scintillation detectors for high‐energy beam dosimetry: I. physical characteristics and theoretical considerations. Phys Med Biol. 1992;37:1883–1900. 1438554 10.1088/0031-9155/37/10/006

[5] Andreo P , Saiful Huq M , Westermark M , et al. Protocols for the dosimetry of high‐energy photon and electron beams: a comparison of the IAEA TRS‐398 and previous international codes of practice. international atomic energy agency. Phys Med Biol. 2002;47:3033–3053. 12361209 10.1088/0031-9155/47/17/301

[6] Vatnitsky S , Moyers M , Miller D , et al. Proton dosimetry intercomparison based on the ICRU report 59 protocol. Radiother Oncol. 1999;51:273–279. 10435822 10.1016/s0167-8140(99)00060-2

[7] Pönisch F , Archambault L , Briere TM , et al. Liquid scintillator for 2D dosimetry for high‐energy photon beams. Med Phys. 2009;36:1478–1485. 19544763 10.1118/1.3106390 PMC2736702

[8] Archambault L , Poenisch F , Sahoo N , et al. Verification of proton range, position, and intensity in IMPT with a 3D liquid scintillator detector system. Med Phys. 2012;39:1239–1246. 22380355 10.1118/1.3681948 PMC3292596

[9] Beddar S , Archambault L , Sahoo N , et al. Exploration of the potential of liquid scintillators for real‐time 3D dosimetry of intensity modulated proton beams. Med Phys. 2009;36:1736–1743. 19544791 10.1118/1.3117583 PMC2832031

[10] Saint‐Gobain Crystals . Organic scintillation materials. USA. 2011 http://www.crystals.saint-gobain.com/.

[11] Robertson D , Hui C , Archambault L , Mohan R , Beddar S . Optical artefact characterization and correction in volumetric scintillation dosimetry. Phys Med Biol. 2014;59:23–42. 24321820 10.1088/0031-9155/59/1/23 PMC3923433

[12] Lacroix F , Beaulieu L , Archambault L , Sam Beddar A . Simulation of the precision limits of plastic scintillation detectors using optimal component selection. Med Phys. 2010;37:412. 20229849 10.1118/1.3276734 PMC2814832

[13] Birks JB . The Theory and Practice of Scintillation Counting. Oxford:Pergamon; 1964

[14] Wook D , Kim LY , Jungwook S , et al. A dose verification method for proton therapy by using a plasticscintillation plate. J Korean Phys Soc. 2009;55:702.

[15] Knoll GF . Radiation Detection and Measurement. 4th Edition. John Wiley & Sons; 2010.

[16] Badhwar GD , Deney CL , Dennis BR , Kaplon MF . The non‐linear response of the plastic scintillator NE102. Nucl Instrum Methods. 1967;57:116–120.

[17] Torrisi L . Plastic scintillator investigations for relative dosimetry in proton therapy. Nucl Instrum Methods Phys Res. 2000;170:523–530.

[18] Wang LLW , Perles LA , Archambault L , Sahoo N , Mirkovic D , Beddar S . Determination of the quenching correction factors for plastic scintillation detectors in therapeutic high‐energy proton beams. Phys Med Biol. 2012;57(23):7767–81. 23128412 10.1088/0031-9155/57/23/7767 PMC3849705

[19] Agostinelli S , Allison J , Amako K , et al. GEANT4 ‐ A simulation toolkit. Nucl Instrum Methods Phys Res, Sect A. 2003;506:250–303.

[20] Allison J , Amako K , Apostolakis J , et al. Geant4 developments and applications. IEEE Trans Nucl Sci. 2006;53:270–278.

[21] Allison J , Amako K , Apostolakis J , et al. Recent developments in GEANT4. Nucl Instrum Methods Phys Res, Sect A. 2016;835:186–225.

[22] Ciocia F , Braem A , Chesi E , et al. GEANT4 studies on the propagation and detection of scintillation light in long thin YAP crystals. Nucl Instrum Methods Phys Res, Sect A. 2009;600:506–512.

[23] Riggi S , La RP , Leonora E , et al. Geant4 simulation of plastic scintillator strips with embedded optical fibers for a prototype of tomographic system. Nucl Instrum Methods Phys Res, Sect A. 2010;624 . Elsevier: 583–590.

[24] Kacperek A . Protontherapy of eye tumours in the UK: a review of treatment at clatterbridge. Appl Radiat Isot. 2009;67:378–386. 18675550 10.1016/j.apradiso.2008.06.012

[25] Helo Y , Kacperek A , Rosenberg I , Royle G , Gibson AP . The physics of cerenkov light production during proton therapy. Phys Med Biol. 2014a;59:7107–7123. 25365447 10.1088/0031-9155/59/23/7107

[26] Helo Y , Rosenberg I , D'Souza D , et al. Imaging cerenkov emission as a quality assurance tool in electron radiotherapy. Phys Med Biol. 2014b;59:1963–1978. 24694567 10.1088/0031-9155/59/8/1963

[27] Coffin D . Decoding raw digital photos. 2000 https://www.cybercom.net/~dcoffin/dcraw/.

[28] Cirrone G , Cuttone G . Hadrontherapy: a geant4‐based tool for proton/ion‐therapy studies. Prog Nucl Sci. 2011;2:207–212.

[29] Guan F , Peeler C , Bronk L , et al. Analysis of the track‐ and dose‐averaged LET and LET spectra in proton therapy using the geant4 Monte Carlo code. Med Phys. 2015;42:6234–6247. 26520716 10.1118/1.4932217 PMC4600086

[30] Meroli S . Energy loss calculator program. 2012 http://meroli.web.cern.ch/meroli/EnergyLossCalculation.html.

[31] Ziegler J , Biersack J , Ziegler M , et al. SRIM 2013 Code 1984–2013. 2013 http://www.srim.org/.‐

[32] ICRU . Stopping Power and Ranges for Protons and Alpha, ICRU Report No. 49. Bethesda MD: ICRU; 1993.

[33] Venselaar J , Welleweerd H , Mijnheer B . Tolerances for the accuracy of photon beam dose calculations of treatment planning systems. Radiother Oncol. 2001;60:191–201. 11439214 10.1016/s0167-8140(01)00377-2

[34] Petric MP , Robar JL , Clark BG . Development and characterization of a tissue equivalent plastic scintillator based dosimetry system. Med Phys. 2006;33:96. 16485414 10.1118/1.2140118

