# Corrigendum

**DOI:** 10.1002/acm2.13335

**Published:** 2021-06-24

In the published article^1^ *J Appl Clin* *Med Phys*, 18: 210–219 (2017) https://doi.org/10.1002/acm2.12143, one affiliation of author Mansour Almurayshid is missing. The additional affiliation for Mansour Almurayshid is “King Abdulaziz City for Science and Technology (KACST), Nuclear Science Research Institute, Riyadh, Saudi Arabia.” This was the affiliation of the author at the time of the original publication and remains the affiliation now. The authors regret the error.
